# An Inventory of Anthelmintic Plants across the Globe

**DOI:** 10.3390/pathogens12010131

**Published:** 2023-01-13

**Authors:** Haroon Ahmed, Seyma Gunyakti Kilinc, Figen Celik, Harun Kaya Kesik, Sami Simsek, Khawaja Shafique Ahmad, Muhammad Sohail Afzal, Sumaira Farrakh, Waseem Safdar, Fahad Pervaiz, Sadia Liaqat, Jing Zhang, Jianping Cao

**Affiliations:** 1 Department of Biosciences, COMSATS University Islamabad (CUI), Park Road, Chakh Shazad, Islamabad 45550, Pakistan; 2 Department of Parasitology, Faculty of Veterinary Medicine, Bingol University, Bingol 12000, Turkey; 3 Department of Parasitology, Faculty of Veterinary Medicine, University of Firat, Elazig 23119, Turkey; 4 Department of Botany, University of Poonch Rawalakot, Azad Jammu and Kashmir 12350, Pakistan; 5 Department of Chemistry, University of Management & Technology (UMT), Lahore 54770, Pakistan; 6 Department of Biological Sciences, National University of Medical Sciences (NUMS), Rawalpindi 46000, Pakistan; 7 The School of Global Health, Chinese Center for Tropical Diseases Research, Shanghai Jiao Tong University School of Medicine, Shanghai 200240, China; 8 National Institute of Parasitic Diseases, Chinese Center for Disease Control and Prevention (Chinese Center for Tropical Diseases Research), Shanghai 200025, China; 9 Key Laboratory of Parasite and Vector Biology, National Health Commission of the People’s Republic of China, Shanghai 200025, China; 10 World Health Organization Collaborating Center for Tropical Diseases, Shanghai 200025, China

**Keywords:** ethnomedicine, anthelmintic, medicinal plant, helminth, global distribution

## Abstract

A wide range of novelties and significant developments in the field of veterinary science to treat helminth parasites by using natural plant products have been assessed in recent years. To the best of our knowledge, to date, there has not been such a comprehensive review of 19 years of articles on the anthelmintic potential of plants against various types of helminths in different parts of the world. Therefore, the present study reviews the available information on a large number of medicinal plants and their pharmacological effects, which may facilitate the development of an effective management strategy against helminth parasites. An electronic search in four major databases (PubMed, Scopus, Web of Science, and Google Scholar) was performed for articles published between January 2003 and April 2022. Information about plant species, local name, family, distribution, plant tissue used, and target parasite species was tabulated. All relevant studies meeting the inclusion criteria were assessed, and 118 research articles were included. In total, 259 plant species were reviewed as a potential source of anthelmintic drugs. These plants can be used as a source of natural drugs to treat helminth infections in animals, and their use would potentially reduce economic losses and improve livestock production.

## 1. Introduction

Livestock production plays a key role in the economic development of a country. Helminthiasis caused by a helminth infection is a major constraint in global livestock production. The mortality and morbidity in animal populations owing to infections caused by parasitic helminths are rapidly increasing worldwide [1]. These parasitic worms are categorized into two major groups: roundworms (phylum Nematoda) and flatworms (phylum Platyhelminthes) [2]. Among these parasites, gastrointestinal parasites pose a serious threat to livestock production. In recent decades, continuous and intensive use of synthetic anthelmintics has been the only method to control gastrointestinal nematodes. However, resistance to all available anthelmintic drug classes has been reported in livestock species. Resistance to an anthelmintic drug is often observed within a few years of introduction of the drug, indicating a remarkably high rate of resistance development, which likely results from a combination of large, genetically diverse parasite populations, and strong selection pressure for resistance. Plants are an ideal source of naturally occurring compounds that can be used as alternative dewormers in livestock [3]. Recently, some anthelmintics have demonstrated loss of efficacy owing to anthelmintic resistance [4]; as a result, parasitic load progressively increases, leading to high mortality and morbidity. Traditional use of medicinal plants for controlling helminth infections is more acceptable owing to the eco-friendly nature and sustainable supply of medicinal plants [5].

**Figure 1:**
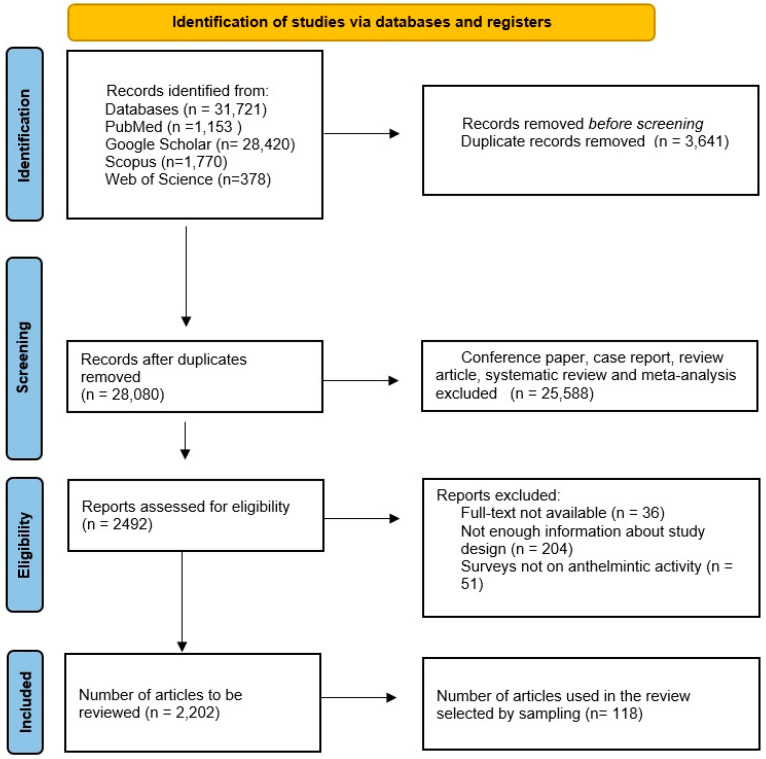
The PRISMA chart showing the summary of the literature search and query results.

The present review is a comprehensive approach to show a geographical distribution of medicinal plants in a given time period and their anthelmintic potential, which would facilitate their use as an effective management strategy against helminth parasites. An electronic search in four major databases (PubMed, Scopus, Web of Science, and Google Scholar) was performed for data published between January 2003 and April 2022. Using database-specific strings, different combinations of the following keywords were used: “anthelmintic activity of plants”, “gastrointestinal nematodes”, “Platyhelminthes”, “roundworms”. The studies were required to include information about plant species, local name, plant family, distribution, plant tissue used, and target parasite species. The PRISMA (Preferred Reporting Items for Systematic Reviews and Meta-Analyses) statement [6] was used as a guide. Prespecified outcome-specific quality criteria were used to judge the admission of each qualitative and quantitative outcome into the appropriate analysis. Two investigators independently reviewed each eligible study and extracted the information and data necessary to carry out the qualitative analysis and the meta-analysis. Disagreements were resolved by consensus among all authors. All relevant studies meeting the criteria were assessed. In some references, multiple lines were used to show them because the authors were working on multiple plant species in the same article. In total, 2202 articles were obtained. However, since not all of them could be included in the current review, it was reduced to 118 articles by sampling (by paying attention to different countries and different plant species and parasites) and used in this review (Figure 1). Finally, 259 plant species from 36 countries worldwide were reviewed as a potential source of anthelmintic drugs. The distribution of the articles used in this review by country is shown in Figure 1. 

The details of anthelmintic plants and their extracts potentially effective against Platyhelminthes and Nematoda are presented in Table 1 and Table 2, respectively.

## 2. Chemical Compounds

The literature review revealed that active chemical compounds present in plants were determined using plant volatile essential oils or extracts in ethanol, butanol, methylene chloride, methanol, hydroalcoholic solvents, dichloromethane, chloroform, petroleum ether, or n-hexane. The following active compounds and secondary metabolites were reported: glycosides, tetrahydroharmine, tannins, gallocatechin, epigallocatechin monomers, jacalin, phytohemagglutinin E2L2, phytohemagglutinin L4, phytohemagglutinin E3L, kidney bean albumin, *Maclura pomifera* agglutinin, *Robinia pseudoacacia* agglutinin, wheat germ agglutinin, cysteine proteinases, ursolic acid, galactolipid 2 and 3, aporphines, hexylresorcinol, *Dolichos biflorus* agglutinin, *Galanthus nivalis* agglutinin, polycarpol, 3-O-acetyl aleuritolic acid, jacalin (jackfruit agglutinin), concanavalin A (jack bean lectin), *Maackia amurensis* lectin, dichloromethane, and plumbagin (Table 3).

## 3. Effect of Plant Extracts in Drug-Resistant Helminths 

Medicinal plant extracts have long been used against helminth parasites in humans and livestock; however, scientific support for their application and research on the characterization of active composites remains limited [123]. Numerous studies have investigated anthelmintic resistance, especially in small ruminants. Most studies have used the fecal egg count reduction test (FECRT), which is based on field management practices. Nevertheless, in vivo experiments on drug efficacy have been conducted in areas with high economic importance. Notably, sheep have been studied more extensively than other livestock species, and a broad spectrum of therapeutics have already been developed for sheep [126].

Molecular methods are promising strategies for in vivo and in vitro diagnosis of many infections and may prove to be effective in the detection of parasitic nematodes and anthelmintic resistance [127,128,129,130]. Gaining knowledge about the mechanisms of resistance will ultimately help to reduce anthelmintic drug resistance in parasites. The diagnosis of drug resistance associated with genomic changes using molecular techniques would help in avoiding unnecessary treatments and thus reduce health complications. However, the use of natural plant compounds has the potential to be a complementary control option that can reduce dependence on drug therapy and delay the development of resistance [127,129,131].

In general, many plant secondary metabolites including chalcones, coumarins, terpenoids, tannins, alkaloids, antioxidants, and flavonoids [132,133] possess anthelmintic and neurotoxic properties [134] and inhibit mitochondrial oxidative phosphorylation [135,136]. These plant-based compounds typically show higher biological activity than synthetic compounds [137]. In many parts of the world, plants have been used for many generations and are still being used to treat parasitic diseases [138]. The identification of novel compounds from plants as anthelmintics is an emerging field of research. According to a study, between 2000 and 2019, 40 patents were granted for natural-product-based nematicides divided into seven structural classes [139], but none of them have yet been commercialized. However, difficulties in determining the mechanism of action of the main active ingredients in plant extracts are among the main barriers for researchers.

## 4. Advantages and Disadvantages of Using Plants for Helminth Parasite Control

Limited information is available on gastrointestinal helminth infections in livestock, which remain a major constraint to livestock production worldwide. Nevertheless, a recent study suggests that anthelmintic plants can be used as a potential resource to improve livestock production [38]. The use of plants as anthelmintics has certain benefits over contemporary veterinary treatments, including affordability, lack of adverse effects, and easy accessibility.

Although most of the information available about the antiparasitic properties of medicinal plants is oral and lacked scientific validity until recently, there is now a growing number of controlled laboratory experiments aiming to confirm and quantify anthelmintic plant activity [24]. Plants can be used in the following two manners: 1. plant parts can be used to cure infected animals naturally or 2. plant extracts and concoctions can be tested both in vitro and in vivo for their anthelmintic potential. The advantages of using antiparasitic plants include effectiveness against species resistant to synthetic anthelmintic drugs, limited or no risk of resistance development, and environmentally friendly procedure [42]. A major drawback is that, to date, only a small number of anthelmintic compounds such as macrocyclic lactones, cyclic octadepsipeptides, benzimidazoles, and imidazothiazoles have been identified in plants after decades of research [65]. Another drawback is the inconsistency between in vitro and in vivo studies on the use of plants as anthelmintics, raising questions regarding their validity and reliability [67]. Additionally, neurological effects associated with the dosage and bioavailability of some medicinal plants need to be elucidated before their use. The choice of an appropriate host–parasite system is tricky in in vivo studies because caring for the animal models adequately is expensive, time-consuming, and labor-intensive [100]. Other drawbacks include uncertainty about plant efficacy, nonspecific responses, irreproducible preparations, and potential negative consequences. An alternative strategy is to use plant secondary metabolites with anthelmintic activity [73]. Secondary metabolites exhibit various modes of action for anthelmintic activity. For example, tannins hinder the feeding process of parasites through forming complexes with parasite proteins or deactivating key enzymes [73]. Terpenes block the tyramine receptors of parasites, whereas alkaloids create unfavorable conditions in the host intestine by generating nitrated and free sugars [97,124]. However, it is important to conduct more studies on the underlying molecular mechanisms and adverse effects on the host to improve drug development.

## 5. Recommendations

An ideal anthelmintic agent should have a broad spectrum of action, a high treatment rate with a single therapeutic dose, low toxicity to the host, and cost-effectiveness. Most currently used synthetic drugs do not meet these requirements. Commonly used drugs have side effects such as nausea, drowsiness, and intestinal disorders. The development of resistance to existing drugs in parasites and the high cost of drugs have led researchers to explore novel anthelmintic effective agents. Ethnobotanical drugs are the source of easily available and effective anthelmintic agents for humans, especially in tropical and developing countries. Thus, people use various herbs or products derived from plants to treat helminth infections. Plants produce secondary metabolites with various ecophysiological functions, such as defense against pathogen attacks and protection against abiotic stresses. These metabolites have potential medicinal effects in humans and animals. 

## 6. Conclusions and Future Perspectives

It is estimated that more than 2.5 billion people are affected with helminth parasites at some stage in their lives. Parasitic diseases remain the major reason of substantial economic loss owing to their impact on livestock health and unexpected deworming costs. According to the literature review, potential anthelmintic plants exhibit great diversity in terms of species and compounds. Nevertheless, initially, all anthelmintics are tested in livestock before being used for human therapy; thus, developments in veterinary anthelmintics could also lead to advancements in human therapy. In addition, studies on nutritional support and vaccination are also required to develop livestock with low parasite susceptibility. 

## Figures and Tables

**Table 1:** List of anthelmintic plants and their extracts effective against flatworms (Platyhelminthes).

Parasite	Study Model	Plant Family	Plant Name	Plant Tissue	Extract	Effective Concentration and Mortality Rate (%)	Reference
*Carmyerius spatiosus*	In vitro	Leguminosae	*Cassia siamea*	Leaves and heartwood	Ethyl acetate extracts	Highest anthelminthic effect	[7]
		Plumbaginaceae	*Plumbago zeylanica*	Roots	n-butanol extract		
		Plumbaginaceae	*Plumbago indica*	Roots	hexane, ethyl acetate, and n-butanol extract		
		Combretaceae	*Terminalia catappa*	Leaves	n-butanol and water extract		
*Clonorchis sinensis*	In vitro	Rosaceae	*Hagenia abyssinica*	Female flowers	Crude extract	5 h (100 µg/mL)	[8]
*Echinococcus granulosus (protoscolex)*	In vitro	Anacardiaceae	*Pistacia atlantica*	Fruits and leaves	Hydroalcoholic extracts	100%; killed protoscoleces (50 mg/mL in 10 min)	[3]
				Leaves and fruits	Hydroalcoholic extracts	0.1% concentration of fresh fruit extract (99.09 ± 1.27 mg/mL) and leaf extract (89.25 ± 18.42 mg/mL) had strong scolicidal effects in 360 min	[9]
	In vitro	Lamiaceae	*Salvia officinalis*	Aerial parts	Ethanolic extract	100% (6–8 days)	[10]
		Fabaceae	*Prosopis farcta*	Leaves	Ethanolic extract Crude alkaloids	25% scolicidal activity with a 500 mg/mL dose after 24 h	[11]
						57% scolicidal activity with a 500 mg/mL dose after 24 h	
		Ranunculaceae	*Nigella sativa*	Seeds	Essential oil (Thymoquinone)	100% scolicidal activity with a 1 mg/mL dose after 10 min	[12]
		Cucurbitaceae	*Dendrosicyos socotrana*	Leaves	Aqueous and methanolic extracts	100% scolicidal activity with a 5000 μg/mL dose after 360 h (methanolic extract) and 408 h (aqueous extract)	[13]
		Euphorbiaceae	*Jatropha unicostata*		Aqueous and methanolic extracts	100% scolicidal activity with a 1000 μg/mL dose after 288 h (both extracts)	
		Berberidaceae	*Berberis vulgaris*	Fruits	Aqueous extracts	98.7% scolicidal activity with a 2 mg/mL dose after 30 min	[14]
		Euphorbiaceae	*Mallotus philippinensis*	Fruits	Methanolic extracts	99% scolicidal activity with a 20 mg/mL dose after 60 min	[15]
*Echinococcus granulosus protoscolex*	In vitro	Meliaceae	*Azadirachta indica*	Whole plant	Ethanolic extracts	Up to 97% mortality with 30 min of incubation	[16]
*Echinostoma caproni*	In vitro	Rosaceae	*Hagenia abyssinica*	Female flowers	Crude extract	51 h (100 µg/mL)	[8]
*Fasciola hepatica*	In vitro	Fabaceae	*Acacia farnesiana*	Leaves	Hexane, ethyl acetate, and methanolic extracts	0% (500 mg/L)	[17]
		Asteraceae	*Artemisia absinthium*			0% (500 mg/L)	
			*Artemisia mexicana*			100% (500 mg/L)	
		Papaveraceae	*Bocconia frutescens*			100% (500 mg/L)	
		Fabaceae	*Cajanus cajan*			100% (500 mg/L)	
		Boraginaceae	*Cordia* spp.			0% (500 mg/L)	
		Malvaceae	*Hibiscus rosa sinensis*			0% (500 mg/L)	
		Verbenaceae	*Lantana camara*			100% (500 mg/L)	
		Fabaceae	*Leucaena diversifolia*			0% (500 mg/L)	
		Meliaceae	*Melia azedarach*			13% (500 mg/L)	
		Lamiaceae	*Mentha* sp.			0% (500 mg/L)	
			*Ocimum basilicum*			0% (500 mg/L)	
		Piperaceae	*Piper auritum*			100% (500 mg/L)	
		*Dysphania*	*Teloxys ambrosioides*			0% (500 mg/L)	
*Fasciola* larvae (sporocyst, redia, and cercaria)	In vitro	Rosaceae	*Potentilla fulgens*	Dried root powder	Ether, chloroform, methanolic, acetone, and ethanolic extracts	8 h LC50 was 54.20 mg/L for sporocysts, 49.37 mg/L for redia, and 38.13 mg/L for cercaria	[18]
*Fasciola gigantica* larvae (sporocysts, redia, and cerceria)	In vivo	Asparagaceae	*Asparagus racemosus*	Dried root powder	Ether, chloroform, methanolic, acetone, and ethanolic extracts	2 h LC50 was 79.93%	[19]
*Fasciola gigantica* and *Taenia solium*	In vitro	Euphorbiaceae	*Acalypha wilkesiana*	Extracts	Methanolic extracts of leaves, stems, and roots	All extracts exhibited anthelmintic activity in vitro	[20]
*Fasciola hepatica*	In vitro	Rosaceae	*Hagenia abyssinica*	Female flowers	Crude extract	1 h (100 µg/mL)	[8]
*Fasciolopsis buski*	In vitro	Zingiberaceae	*Alpinia nigra*	Shoot	Crude alcoholic extract	3.94 ± 0.06 h death time (20 mg/mL concentration)	[21]
*Gastrothylax crumenifer*	In vitro	Fabaceae	*Sesbania sesban var. bicolor*	Fresh leaves	Methanolic extracts of dried plants	Better than praziquantel	[22]
		Cyperaceae	*Cyperus compressus*	Roots			
		Asparagaceae	*Asparagus racemosus*	Roots			
*Hymenolepis diminuta* and *Syphacia obvelata*	In vitro In vivo	*Asparagaceae*	*Asparagus racemosus*	Roots	Methanolic extract	53.88% and 24% reduction in EPG * and worm counts, respectively (30 mg/mL concentration)	[23]
*Hymenolepis diminuta*	In vitro	Cyperaceae	*Cyperus compressus*	Roots	Methanolic extract	61.74% reduction in the EPG and 24% reduction in worm counts (30 mg/mL concentration)	[24]
*Hymenolepis diminuta*	In vitro	Fabaceae	*Sesbania sesban*	Fresh Leaves	Methanolic extract	65.10% reduction in EPG counts, 56% reduction in worm counts (30 mg/mL concentration)	[25]
*Paramphistomum gracile*	In vitro	Fabaceae	*Senna alata*, *S. alexandrina*, and *S. occidentalis*	Leaf extract	Ethanolic extracts	Dose-dependent effects on motility and mortality	[26]
*Paramphistomum microbothrium*	In vitro	Zygophyllaceae	*Balanites aegyptiaca*	Fruits	Methanolic extract	200 µg/ml, at which distinct damage to the whole body surface of the trematodes	[27]
*Raillietina echinobothrida*	In vitro	Asteraceae	*Acmella oleracea*	Leaves	Methanolic extract	18.42 ± 0.95 h survival time (20 mg/mL concentration)	[28]
*Raillietina spiralis*	In vitro	Malvaceae	*Thespesia lampas*	Roots	Aqueous extracts	51 ± 0.33 min death time (20 mg/mL concentration)	[29]
*Raillietina spiralis*	In vitro	Meliaceae	*Azadirachta Indica*	Leaves	Aqueous extract	46 ± 0.53 min death time (20 mg/mL concentration)	[30]
*Raillietina spiralis*	In vitro	Scrophulariaceae	*Verbascum Thapsus*	Fresh Leaves	Methanolic extract	86 ± 5 min death time (20 mg/mL concentration)	[31]
*Raillietina spiralis*	In vitro	Asteraceae	*Achillea wilhelmsii*	Fresh Leaves	Methanolic extract	40 min death time (20 mg/mL concentration)	[32]
*Raillietina spiralis*	In vitro	Lauraceae	*Cinnamomum camphora*	Leaves	Aqueous extracts	47 ± 0.54 min death time (20 mg/mL concentration)	[33]
*Raillietina spiralis*	In vitro	Verbenaceae	*Clerodendron inerme*	Leaves	Aqueous extracts	45 ± 0.52 min death time (20 mg/mL concentration)	[34]
*Raillietina tetragona*	In vitro	Poaceae	*Imperata cylindrica*	Underground parts (rhizomes and roots)	Chloroform (medium polar solvent)	Dose-dependent anthelmintic activity	[35]
*Schistosoma mansoni*	In vitro	Apocynaceae	*Rauwolfia vomitoria*	Stem bark and roots	Ethanolic extract	High activity against cercariae and adult worms	[36]
*Syphacia obvelata*	In vitro	Cyperaceae	*Cyperus compressus*	Roots	Methanolic extract	28.92% reduction in the EPG and 33.85% reduction in worm counts (30 mg/mL concentration)	[24]
*Syphacia obvelata*	In vitro	Fabaceae	*Sesbania sesban*	Fresh leaves	Methanolic extract	EPG and worm counts reduced by 34.32% and 47.08%, respectively (30 mg/mL concentration)	[25]
*Schistosoma mansoni*	In vivo	Asteraceae	*Baccharis trimera*	Leaves	Crude dichloromethane extract (DE) and aqueous fraction (AF)	98% (AF) 97% (DE)	[37]
			*Tanacetum vulgare*	Aerial parts	Crude extract and Essential oil	100%	[38]
*Schistosoma mansoni*	In vitro	Rosaceae	*Hagenia abyssinica*	Female flowers	Crude extract	3 h (100 µg/mL)	[8]
*Schistosoma mansoni*	In vitro	Euphorbiaceae	*Euphorbia conspicua*	Leaves	Leaf extract	100% (100 µg/mL)	[39]
		Piperaceae	*Piper chaba*	Fruits	Methylene chloride extract	Strongest activity	[40]
*Taenia solium*	In vitro	Asclepiadaceae	*Pergularia daemia*	Leaves	Ethanolic extract	210.00 ± 0.52 min death time (25 mg/mL concentration)	[41]
					Aqueous extract	221.12 ± 0.61	
*Taenia tetragona*	In vitro	Asteraceae	*Acmella oleracea*	Leaves	Hexane extract	The lethal concentration (LC50) of the plant extract was 5128.61 ppm on *T. tetragona* and 8921.50 ppm on *A. perspicillum*	[42]

* EPG: Egg per gram.

**Table 2:** List of anthelmintic plants and their extracts effective against roundworms (Nematoda).

Parasite	Study Model	Plant Family	Plant Name	Plant Part Used	Extract/Compound	LC50 *	References
*Allolobophora caliginosa*	In vitro	Fabaceae	*Indigofera oblongifolia*	Leaves	Leaf extracts	15 ± 2 and 8.6 ± 1 h survival time with leaf extracts at 200 mg/mL and 300 mg/mL, respectively	[43]
*Ancylostoma caninum*, *Haemonchus placei*, andCyathostomins	In vivo	Ebenaceae	*Diospyros anisandra*	Leaves and bark	Extracts and active compounds	Wide-spectrum anthelmintic activity	[44]
*Ascardia galli*	In vitro	Malvaceae	*Thespesia lampas*	Roots	Aqueous extracts	43 ± 0.86 min death time (20 mg/mL concentration)	[29]
*Ascardia galli*	In vitro	Mimosaceae	*Acacia oxyphylla*	Fresh stems	Ethanolic extracts	55.17 h ± 1.04 h death time (0. 5 mg/ mL concentration)	[45]
*Ascardia galli*	In vitro	Meliaceae	*Azadirachta Indica*	Leaves	Aqueous extract	46 ± 0.26 min death time (20 mg/mL concentration)	[30]
*Ascardia galli*	In vitro	Scrophulariaceae	*Verbascum Thapsus*	Fresh Leaves	Methanolic extract	81 ± 4 min death time (20 mg/mL concentration)	[31]
*Ascardia galli*	In vitro	Asteraceae	*Achillea wilhelmsii*	Fresh Leaves	Methanolic extract	40 min death time (20 mg/mL concentration)	[32]
*Ascardia galli*	In vitro	Lauraceae	*Cinnamomum camphora*	Leaves	Aqueous extracts	52 ± 0.43 min death time (20 mg/mL concentration)	[33]
*Ascardia galli*	In vitro	Verbenaceae	*Clerodendron inerme*	Leaves	Aqueous extracts	50 ± 0.31 min death time (20 mg/mL concentration)	[34]
*Ascardia galli* and *Pheretima posthuma*	In vitro	Malvaceae	*Malvastrum coromandelianum*	Leaves	Methanolic and ethyl acetate extracts	Significant anthelmintic activity	[46]
*Ascaris lumbricoides*	In vitro	Musaceae	*Musa paradisiaca*, *M. sapientum*, *and M. nana*	Roots	Methanol root extracts	Death time 151.39 ± 0.1 min at 200 mg/mL	[47]
*Ascaris lumbricoides*	In vitro	Asclepiadaceae	*Pergularia daemia*	Leaves	Ethanolic Extract	98.42 ± 0.57 min death time (25 mg/mL concentration)	[41]
					Aqueous Extract	109.91 ± 0.49 min death time (25 mg/mL concentration)	
*Ascaris suum L3 larvae*	In vitro	Lythraceae	*Punica granatum*	Fruit Peel	Ethanolic extracts	EC50 values 164%	[48]
		Rutaceae	*Zanthoxylum zanthoxyloides*	Roots		EC50 values 97%	
		Rutaceae	*Clausena anisata*	Roots		EC50 values 74%	
*Ascaris suum L3 larvae*	In vitro				Acetone/water extracts	*Ascaris suum L3* migratory inhibition activity EC50 ** values	[49]
		Pinaceae	*Pinus sylvestris*	Bark		48.2%	
		Fabaceae	*Onobrychis viciifolia*	Whole plant		41.9%	
		Fabaceae	*Trifolium repens*	Flowers		98.4%	
		Grossulariaceae	*Ribes nigrum*	Leaves		91.8%	
			*Ribes rubrum*	Leaves		86%	
*Brugia malayi*	In vivo	Piperaceae	*Piper betle*	Leaves	Methanolic extracts	Moderate activity	[50]
*Brugia malayi*	In vitro/ In vivo	Apiaceae	*Trachyspermum ammi*	Dried fruits	Methanolic extracts	58.93%	[51]
*Brugia malayi*	In vivo	Caesalpiniaceae	*Caesalpinia bonducella*	Seed kernels	Ethanolic extracts	96.0% microfilaricidal and 100% sterilization in females	[52]
					Butanolic extracts		
					Aqueous fraction		
*Brugia malayi*	In vivo/ In vitro	Verbenaceae	*Lantana camara*	Stem	Ethanolic extracts	43.05% adulticidal activity; sterilization of 76% of surviving females	[53]
*Brugia pahangi*	In vitro	Asteraceae	*Neurolaena lobata*	Leaves	Ethanolic extracts	Completely immotile after 24 h incubation at 500 μg/mL concentration	[54]
*Caenorhabditis elegans*	In vitro	Laminaceae	*Tetradenia riparia*	Leaves	Ethyl acetate extracts	Most effective minimum lethal concentration value was 0.004 mg/mL	[55]
*Caenorhabditis elegans*	In vitro	Combretaceae	*Anogeissus leiocarpus*	Stem bark	Ethanolic extracts	72 h LC50 was between 0.38 and 4.00 mg/mL	[56]
		Meliaceae	*Khaya senegalensis*	Leaves			
		Euphorbiaceae	*Euphorbia hirta*				
		Annonaceae	*Annona senegalensis*		Aqueous extracts		
		Apocynaceae	*Parquetina nigrescens*				
*Caenorhabditis elegans*	In vitro	Sapindaceae	*Acer rubrum*	Leaves	Ethanolic extracts	Killed 50% (LC50) or 90% (LC90) of the nematodes in 24 h	[57]
		Fagaceae	*Quercus alba*				
		Rosaceae	*Rosa multiflora*				
		Anarcardiaceae	*Rhus typhina*				
		Fabaceae	*Robinia pseudoacacia*				
			*Lespedeza cuneata*	Leaves and stems			
*Caenorhabditis elegans*	In vitro	Meliaceae	*Khaya senegalensis*	Stem bark	Ethanolic and aqueous extracts	72 h LC50 was between 0.38 and 4.00 mg/mL	[56]
		Combretaceae	*Anogeissus leiocarpus*	Leaves			
		Euphorbiaceae	*Euphorbia hirta*				
		Annonaceae	*Annona senegalensis*				
		Apocynaceae	*Parquetina nigrescens*				
		Fabaceae	*Senna petersiana*				
*Caenorhabditis elegans*	In vitro	Plumbaginaceae	*Plumbago indica*	Root	Methylene chloride	Strongest activity	[40]
*Cooperia* spp.	In vitro	Fabaceae	*Leucaena leucocephala*	Fresh leaves	Aqueous extract	52.02 ± 12.39 of egg hatching within 48 h of exposure	[58]
*Eudrilus eugeniae*	In vitro	Lamiaceae	*Ocimum basilicum*	Fruits	Ethanol and hexane extracts	213.39 ± 1.05 and 362.98 ± 1.54 death time of ethanolic extract and hexane extract, respectively, at 250 μg/mL concentration	[59]
Gastrointestinal nematodes	In vitro/ In vivo	Lamiaceae	*Prunella vulgaris*	Whole plant	Phenolic compounds	Highest nematode motility (100%) with higher concentrations of methanolic extracts (50 mg/ mL)	[60]
Gastrointestinal nematodes	In vivo	Lythraceae	*Punica granatum*	Fruit peel	Pomegranate peel extract	7 days after the first and second doses, 85–97% decrease in fecal egg count (FEC)	[61]
Gastrointestinal nematodes	In vitro	Moringaceae	*Moringa oleifera* lectin	Seeds	Distilled water homogenization	40.4% of eggs unhatched at 250 μg/mL dose	[62]
Gastrointestinal nematodes	In vitro	Phyllanthaceae	*Bridelia ferruginea*	Leaves	Methanolic and acetone extracts	The number of eggs that hatched was reduced in a concentration-dependent manner (*p* < 0.01) upon treatment	[63]
		Combretaceae	*Combretum glutinosum*				
		Rubiaceae	*Mitragyna inermis*				
Gastrointestinal nematodes of goats	In vitro	Vitaceae	*Cissus quadrangularis*	Aerial parts	Aqueous (cold and boiled) and methanolic extracts	Statistically significant effect	[64]
		Asphodelaceae	*Aloe marlothii*	Leaves			
		Mimosoideae	*Albizia anthelmintica*	Bark			
		Vitaceae	*Cissus rotundifolia*	Bark			
		Anacardiaceae	*Sclerocarya birrea*	Bark			
		Fabaceae	*Vachellia xanthophloea*	Bark			
Gastrointestinal nematodes of sheep	In vivo	Punicaceae	*Punica granatum*	Fruit (seeds and peel)	Boiled extracts	8–40% (21st day)	[65]
		Asteraceae	*Artemisia campestris*	Whole plant		3–36% (21st day)	
		Salicaceae	*Salix caprea*	Bark and leaves		7–40% (21st day)	
Gastrointestinal nematodes of sheep	In vitro	Myrtaceae	*Psidium cattleianum*	Fruits	Hydroalcoholic extract	80% in the inhibition of larval migration	[66]
Gastrointestinal nematodes of sheep	In vitro	Punicaceae	*Aqueous Pomegranate*	Fruit pulp	Methanolic and gallic acid extracts	Significant inhibition of egg hatching within 48 h of exposure, highlighting a high (>82%) efficacy in vitro at all tested doses	[67]
*Gastrothylax crumenifer*	In vitro	Menispermaceae	*Tinospora cordifolia*	Plant stems	Alcoholic and aqueous extracts	mortality rate of 100% at concentration of 100 mg/mL	[68]
*Haemonchus contortus*	In vitro	Asteraceae	*Artemisia maritima*	Whole plants	Methanolic extracts	84.5%	[69]
			*Artemisia vestita*			87.2%	
*Haemonchus contortus*	In vitro	Ericaceae	*Arctostaphylos uva-ursi*	Leaves	Methanolic extracts	95.8 ± 0.5% inhibition in DMSO	[70]
		Anacardiaceae	*Rhus glabra*			90.2 ± 0.9% inhibition in DMSO	
		Asteraceae	*Balsamorhiza sagittata*			88.1 ± 1.2% inhibition in DMSO	
		Ranunculaceae	*Caltha palustris*			86.5 ± 1.2% inhibition in DMSO	
		Boraginaceae	*Cynoglossum officinale*			84.7 ± 1.0% inhibition in DMSO	
		Asteraceae	*Solidago mollis*			82.8 ± 1.4% inhibition in DMSO	
		Asteraceae	*Centaurea stoebe*			78.1 ± 1.5% inhibition in DMSO	
		Fabaceae	*Glycyrrhiza lepidota*			77.6 ± 2.3% inhibition in DMSO	
		Anacardiaceae	*Rhus aromatica*			100% inhibition in DMSO	
		Asteraceae	*Ericameria nauseosa*			100% inhibition in DMSO	
		Apiaceae	*Perideridia gairdneri*			100% inhibition in DMSO	
		Geraniaceae	*Geranium viscosissimum*			100% inhibition in DMSO	
		Asteraceae	*Chrysothamnus viscidiflora*			100% inhibition in DMSO	
		Asteraceae	*Liatris punctata*	Roots		100% inhibition in DMSO	
		Fabaceae	*Melilotus alba*	Leaves		100% inhibition in DMSO	
		Fabaceae	*Melilotus officinalis*			100% inhibition in DMSO	
		Papaveraceae	*Sanguinaria canadensis*	Roots		98.5 ± 0.3% inhibition in DMSO	
		Orobanchaceae	*Pedicularis racemosa*	Leaves		74.2 ± 0.9% inhibition in DMSO	
		Lamiaceae	*Stachys palustris*			72.9 ± 1.8% inhibition in DMSO	
		Lamiaceae	*Agastache foeniculum*			70.05 ± 0.7% inhibition in DMSO	
		Lamiaceae	*Monarda fistulosa*			69.5 ± 1.5% inhibition in DMSO	
		Fabaceae	*Pediomelum argophyllum*			69.7 ± 1.8% inhibition in DMSO	
		Lamiaceae	*Lycopus americanus*			76.0 ± 2.3% inhibition in DMSO	
		Ranunculaceae	*Clematis ligusticifolia*			68.7 ± 2.0% inhibition in DMSO	
		Amaryllidaceae	*Allium cernuum*			68.4 ± 1.3% inhibition in DMSO	
		Asteraceae	*Conyza canadensis*			76.8 ± 2.1% Inhibition in MOPS	
		Cornaceae	*Cornus sericea*			57.4 ± 3.1% inhibition in DMSO	
		Rosaceae	*Rubus idaeus*			51.9 ± 1.6% inhibition in DMSO	
		Ranunculaceae	*Actaea rubra*			45.2 ± 1.5% Inhibition in DMSO	
		Caprifoliaceae	*Symphoricarpos occidentalis*			43.1 ± 3.3% Inhibition in DMSO	
		Asteraceae	*Artemisia ludoviciana*			40.8 ± 2.0% inhibition in DMSO	
		Asteraceae	*Artemisia frigida*			36.2 ± 1.65% inhibition in DMSO	
		Asteraceae	*Tanacetum vulgare*			33.5 ± 2.0% inhibition in DMSO	
		Cleomaceae	*Cleome serrulata*			23.9 ± 1.7% Inhibition in DMSO	
		Onagraceae	*Epilobium angustifolium*			23.2 ± 3.5% inhibition in DMSO	
		Fagaceae	*Quercus macrocarpa*			18.3 ± 2.2% Inhibition in DMSO	
		Salicaceae	*Salix exigua*			5.9 ± 0.7% Inhibition in DMSO	
*Haemonchus contortus*	In vitro	Asteraceae	*Artemisia absinthium*	Leaves	Crude aqueous and ethanolic extracts	Aqueous extracts exhibited greater anthelmintic activity	[71]
*Haemonchus contortus*	In vitro	Rutaceae	*Citrus aurantifolia*	Essential oils from fruit peel	Oil extracts	Oil has limonene (56.37%), β-pinene (11.86%) and γ-terpinene (11.42%)	[72]
		Annonaceae	*Annona muricata*	Leaves	Aqueous extracts	Aqueous extract of A. muricata leaves at serial dilutions of 50%, 25%, 12.5% and 6.25% inhibited the motility of L3 by 83.29%, 89.08%, 74.62% and 30.47% respectively	
*Haemonchus contortus*	In vitro	Anacardiaceae	*Myracrodruon urundeuva*	Seeds	Ethanolic and hexane extracts	Inhibition of larval development (LC50 = 0.29 mg mL^−1^)	[73]
*Haemonchus contortus*	In vitro	Liliaceae	*Allium sativum*	Bulbs	Ethanolic extracts	84.0 ± 4.3	[74]
		Asphodelaceae	*Aloe ferox*	Leaves		86.9 ± 2.9	
		Bromeliaceae	*Ananas comosus*			100 ± 1.0	
		Caricaceae	*Carica papaya*			76.0 ± 5.1	
		Moraceae	*Ficus benjamina*			78.1 ± 3.5	
		Moraceae	*Ficus ingens*			78.1 ± 5.7	
		Moraceae	*Ficus carica (brown)*			56.3 ± 2.8	
		Moraceae	*Ficus carica (white)*			74.1 ± 7.9	
		Moraceae	*Ficus indica*			44.5 ± 7.0	
		Moraceae	*Ficus lutea*			60.0 ± 6.3	
		Moraceae	*Ficus elastica*			77.8 ± 6.6	
		Moraceae	*Ficus natalensis*			68.8 ± 7.2	
		Moraceae	*Ficus sur*			81.3 ± 5.6	
		Moraceae	*Ficus sycomorus*			6.3 ± 4.3	
		Moraceae	*Ficus ornamental thai*			60.0 ± 1.7	
		Lamiaceae	*Leonotis leonurus*			56.5 ± 6.1	
		Moraceae	*Melia azedarach*			66.7 ± 4.4	
		Fabaceae	*Peltophorum africanum*			65.2 ± 4.0	
		Amaryllidaceae	*Scadoxus puniceus*			59.4 ± 8.2	
		Fabaceae	*Lespedeza cuneata*			100 ± 1.6	
		Leguminosae	*Tephrosia inandensis*			64.0 ± 7.8	
		Canellaceae	*Warburgia ugandensis*			81.5 ± 3.5	
		Canellaceae	*Warburgia salutaris*			80.8 ± 3.4	
		Cucurbitaceae	*Cucumis myriocarpus*			60.0 ± 5.7	
		Zingiberaceae	*Zingiber officinale*	Rhizomes		72.0 ± 2.5	
*Haemonchus contortus*	In vitro	Asteraceae	*Vernonia amygdalina*	Leaves	Hot water extracts	Ineffective	[75]
		Annonaceae	*Annona senegalensis*	Stem barks		88.5%	
*Haemonchus contortus*	In vivo	Fabaceae	*Acacia nilotica*	Leaves	Without extraction	10% reduction in worm	[76]
			*Acacia karroo*			34% reduction in worm	
*Haemonchus contortus*	In vitro and In vivo	Amaranthaceae	*Chenopodium ambrosioides*	Leaves and stems	Organic maceration	96.3% (invitro), 45.8% (in vivo) at 40 mg/mL dose	[77]
		Simaroubaceae	*Castela tortuosa*			78.9% (in vitro) 27.1% (in vivo) at 20 mg/mL dose	
*Haemonchus contortus*	In vivo and In vitro	Lamiaceae	*Mentha pulegium*	Aerial parts	Hydroethanolic extract	91.58% inhibition in the egg hatch assay at 8 mg/mL after 48 h. 65.2% inhibition at 8 mg/mL after 8 h in adult worm motility	[78]
*Haemonchus contortus*	In vitro	Apocynaceae	*Tylophora Indica*	Leaves	Methanolic extract	100% mortality after 6 h exposure at 50 mg/mL of concentration	[79]
*Haemonchus contortus*	In vitro	Passifloraceae	*Turnera ulmifolia*	Leaves and roots	Hydroacetonic and hydroalcoholic extracts	The highest egg hatching inhibition with the lowest LC50 value of 430 μg/mL (95%, CI 400–460 μg/mL)	[80]
		Fabaceae	*Parkia platycephala*	Leaves and seeds		LC50 1340, 95% CI 1170-1550 μg/mL	
		Fabaceae	*Dimorphandra gardneriana*	Leaves and bark		Ineffective	
*Haemonchus contortus*	In vitro	Lauraceae	*Persea americana*	Dried seeds	Hot water extracts	76.9 ± 7.2% effective in 500 μg/mL dose	[81]
*Haemonchus contortus*	In vitro and In vivo	Asteraceae	*Artemisia absinthium*	Whole plant	Crude methanolic extracts	Strong anthelmintic effect	[82]
		Malvaceae	*Malva sylvestris*				
*Haemonchus contortus*	In vitro	Asteraceae	*Artemisia herba-alba*	Stems and leaves	Crude methanolic extracts	98.67% inhibition of egg hatching at 1 mg/mL concentration	[83]
		Punicaceae	*Punica granatum*	Peel and roots		Eggs unhatched at the end of the observation period	
*Haemonchus contortus*	In vitro	Asteraceae	*Artemisia vulgaris*	Leaves	Aqueous and ethanolic extracts	%100	[84]
*Haemonchus contortus*	In vitro	Fagaceae	*Castanea sativa*	Stems and leaves	Ethanolic extracts	All plants showed some anthelmintic activity on both L3 larvae and adult worms)	[85]
		Fabaceae	*Sarothamnus scoparius*	Stems and leaves			
		Pinaceae	*Pinus sylvestris*	Stems and leaves			
		Fagaceae	*Quercus robur*	Leaves			
		Oleaceae	*Fraxinus excelsior*	Leaves			
		Betulaceae	*Corylus avellana*	Leaves			
		Ericaceae	*Erica erigena*	Stems and leaves			
		Fabaceae	*Acacia holosericea*				
			*Acacia salicina*				
		Cupressaceae	*Callitris endlicheri*				
			*Casuarina cunninghamiana*				
		Lauraceae	*Neolitsea dealbata*				
*Haemonchus contortus*	In vivo	Asteraceae	*Artemisia absinthium*	Whole plant	Aqueous and methanolic extracts	4.3–67.2% reduction in EPG	[86]
*Haemonchus contortus*	In vitro	Asteraceae	*Artemisia absinthium*	Aerial parts	Crude aqueous extracts	Worm motility inhibition was 73.6%	[87]
					Crude ethanolic extracts	Worm motility inhibition was 94.7%	
*Haemonchus contortus*	In vivo	Anacardiaceae	*Pistacia lentiscus*	Leaves	Acetone extracts	Significant decreases in egg excretion	[88]
		Fagaceae	*Quercus coccifera*				
			*Onobrychis viciifolia*				
			*Ceratonia siliqua*				
			*Medicago sativa*				
*Haemonchus contortus* eggs	In vitro	Combretaceae	*Terminalia glaucescens*	Leaves	Methanolic extracts	87.55% inhibition of egg hatching at the 100 µg/mL dose	[89]
*Haemonchus contortus* eggs	In vitro	Lamiaceae	*Leucas martinicensis*	Stems and bark	Crude aqueous and hydroalcoholic extracts	Complete inhibition of egg hatching at the 1 mg/mL dose	[90]
			*Leonotis ocymifolia*	Aerial parts			
		Fabaceae	*Senna occidentalis*	Leaves			
		Polygonaceae	*Rumex abyssinicus*	Stems and bark			
		Leguminosae	*Albizia schimperiana*				
*Haemonchus contortus* eggs and larvae	In vitro	Fabaceae	*Acacia farnesiana*	Dried pods	Hydroalcoholic extracts	100% ovicidal and 75.2% larvicidal activity at the 50 mg/mL dose	[91]
*Haemonchus contortus* eggs and larvae	In vitro	Fabaceae	*Senegalia gaumeri*	Leaves	Methanolic extracts	Ovicidal effect in the morula stage	[92]
*Haemonchus* spp.	In vitro	Casuarinaceae	*Allocasuarina torulosa*	Fresh leaves	Methanolic extracts	64.14–89.83% exposure at the 30 mg/mL concentration	[93]
		Fabaceae	*Acacia holosericea*				
			*Acacia salicina*				
		Cupressaceae	*Callitris endlicheri*				
		Casuarinaceae	*Casuarina cunninghamiana*				
		Lauraceae	*Neolitsea dealbata*				
*Onchocerca gutturosa*	In vitro	Annonaceae	*Polyalthia suaveolens*	Bark	Hexane extracts	Significant inhibitory effect on the vitality of adult male worms	[94]
		Euphorbiaceae	*Discoglypremna caloneura*				
*Onchocerca ochengi*	In vitro	Salicaceae	*Homalium africanum*	Leaves	Hexane methylene chloride extracts	Significant effect	[95]
*Parascaris equorum*	In vitro	Asteraceae	*Artemisia dracunculus*	Leaves	Methanolic extracts	90% inhibition of egg hatching and high larvicidal effect at concentrations ≥100 mg/mL	[96]
		Myrtaceae	*Eucalyptus camadulensis*	Leaves			
		Lamiaceae	*Mentha pulegium*	Aerial parts			
		Lamiaceae	*Zataria multiflora*	Aerial parts			
		Liliaceae	*Allium sativum*	Bulbs			
*Pheretima posthuma*	In vitro	Nyctaginaceae	*Bougainvillea spectabilis*	Crude extract of flowers	Ethanolic and aqueous extracts	39 min (time of death) at a concentration of 50 mg/mL	[97]
*Pheretima posthuma*	In vitro	Acanthaceae	*Barleria buxifolia*	Leaves	Ethanolic extract	89.00 ± 1.82 min for death time at a concentration of 100 mg/mL	[98]
*Pheretima posthuma*	In vitro	Plumbaginaceae	*Plumbago* *zeylanica*	Leaves	Methanolic Extract	81 ± 1.5 min death time (concentration of 20 mg/mL)	[99]
					Water Extract	228 ± 1.2 min death time (concentration of 20 mg/mL	
*Strongyloides venezuelensis*	In vitro	Siparunaceae	*Siparuna guianensis*	Leaves	Hexane extracts	Significant inhibitory effect on the vitality of adult male worms	[100]
*Toxocara vitulorum*	In vitro	Zygophyllaceae	*Balanites aegyptiaca*	Fruits	Methanolic extract	120 μg/ml after 24 h complete disruption of the muscle cells	[101]
*Teladorsagia circumcincta* L1 larvae	In vivo	Fabaceae	*Phaseolus vulgaris*	Seeds	Lectin purification	Worm burden 4416 ± 878 (control) 3475 ± 792 (treated)	[102]
*Trichostrongylus colubriformis* L1 larvae						Worm burden 6708 ± 414 (control) 6500 ± 295.5 (treated)	
*Trichostrongylus colubriformis*	In vivo	Moraceae	*Artocarpus integrifolia*	Whole plant	Ethanolic extracts	Reduced concentration of nematode eggs (2.3 mg semi-purified PHA lectin/kg LW/day)	[102]
		Fabaceae	*Canavalia ensiformis*				
		Fabaceae	*Phaseolus vulgaris*				
		Fabaceae	*Maackia murensis*				
		Fabaceae	*Robinia pseudoacacia*				
		Moraceae	*Maclura pomifera*				
		Fabaceae	*Dolichos biflorus*				
		Poaceae	*Triticum vulgare*				
		Amaryllidaceae	*Galanthus nivalis*				
		Rosaceae	*Rosa multiflora*				

* LC50: Lethal concentration. ** EC50: Effective concentration.

**Table 3:** Candidate natural substances with anthelmintic effects.

Compound	Parasite Species	Study Model	Reported Mortality	Reference
A penta-substituted pyridine alkaloid	*Schistosoma mansoni*	In vitro	100%	[103]
Essential oil	*Echinococcus granulosus (protoscolex)*	In vitro	79.22% scolicidal activity with the 20 mg/mL dose during 60 min	[104]
Essential oil (Thymoquinone)	*Echinococcus granulosus* *(protoscolex)*	In vitro	100% scolicidal activity with the 1 mg/mL dose after 10 min	[12]
Essential oil	*Haemonchus contortus*	In vitro and in vivo	33.3% and 87.5% inhibition motility for flower essential oil	[105]
			29.1% and 75% for leaf essential oil	
			87.2%	
Lectin purification	*Teladorsagia circumcincta (L1)*	In vivo	Worm burden 4416 ± 878 (control) 3475 ± 792 (treated)	[102]
	*Trichostrongylus colubriformis (L1)*		Worm burden 6708 ± 414 (control) 6500 ± 295.5 (treated)	
Tannin	*Cooperia* spp.	In vivo	Higher activity	[106]
Cysteine proteinases (CP)	*Hymenolepis diminuta*	In vitro	CP extracts exhibited anthelmintic activity in vitro	[107]
Pristimerin	*Anticestodal*	Invitro In vivo	EPG by 94 ± 5%, 8 ± 4%, 6 ± 3%, and 97 ± 4%, respectively	[60]
Ursolic acid	*Brugia malayi*	Invitro In vivo	86% inhibition	[108]
Withaferin A	*Brugia malayi*	In vivo	4.3% reduced parasite load using 8 μg/mL within 24 h	[109,110]
Galactolipid-1 Galactolipid-2 Galactolipid-3 Galactolipid-4	*Brugia malayi*	In vitro In vivo	Fraction F1: 80%; Fraction F2: 30%; Fraction F3: 40%; Fraction F4: 100% (31.25 μg/mL)	[111]
Curcumin	*Schistosoma mansoni*	In vitro	100% mortality in male and female	[112]
Aporphine	*Anisakis simplex* and *Hymenolepis nana*	In vitro	No cestocidal and nematocidal effects against *H. nana* and *A. simplex*	[113]
Derived saponins	Donkey Gastrointestinal Nematodes	In vitro	Significant (*p* < 0.05) inhibition of nematode egg hatching (>80%)	[114]
Maclura pomifera agglutinin	*Teladorsagia circumcincta*	In vivo	Direct anthelmintic effect on nematode fecundity and an indirect effect by enhancing local immune responses in the host	[102]
Tannins	*Teladorsagia circumcincta*, *Haemonchus contortus*, and *Trichostrongylus colubriformis*	In vitro	Larval migration inhibition assay on third-stage larvae (L3) and adult worm motility inhibition assay	[85]
Essential oil	*Gastrointestinal nematodes*	In vitro	33.30% inhibition motility	[105]
			87.50% inhibition motility	
Saponins	*Gastrointestinal nematodes*	In vitro	Strong anthelmintic activity	[115]
	Donkey strongyles	In vitro	Strong anthelmintic activity	[116]
Tannins	*Trichostrongylus colubriformis*	In vitro	Larval migration inhibition assay on third-stage larvae (L3) and adult worms	[85]
Condensed and hydrolyzable tannins	*Caenorhabditis elegans*	In vitro	Killed 50% (LC50) or 90% (LC90) of nematodes in 24 h	[57]
Tannins	*Trichostrongylus colubriformis*	In vitro	Larval migration inhibition assay on third-stage larvae (L3) and adult worms	[85]
Flavonoids, condensed tannins, and gallotannin	*Caenorhabditis elegans*	In vitro	Minimum lethal concentration was 0.13–0.52 mg/mL	[117]
Methylene chloride	*Caenorhabditis elegans*	In vitro	Strongest effect	[40]
Tannins, phenolic compounds, and steroids	*Haemonchus contortus*	In vitro, In vivo	100% inhibition of egg hatching, highest activity for adult motility, and larvicidal assay	[118]
Antimicrobial agents, alkaloids, flavonoids, tannins, and phenols	*Haemonchus contortus*	In vitro	High activity for adulticidal and egg hatching inhibition	[119]
Polyphenols	*Caenorhabditis elegans*	In vitro and in vivo	Inhibition of larval migration	[120]
Phenolic compounds	*Gastrointestinal nematodes*	In vitro In vivo	Highest nematode motility (100%) in the higher concentrations of methanolic extract (50 mg/mL)	[60]
Presence of saponin, alkaloids, flavonoids, and tannins	*Haemonchus contortus*	In vitro	High mortality rate	[121]
Presence of eugenol and asarone	*Moniezia expansa*	In vitro	100 mg/mL concentration and the time taken for the paralysis of the parasite amounts to 66.3 ± 0.03 min and death was recorded after 93.2 ± 0.09 min	[122]
Proanthocyanidins and flavonoids	*Haemonchus contortus*	In vitro	Larval migration inhibition and adult worms’ motility inhibition	[123]
**Essential oils**	*Neoechinorhynchus buttnerae*, endoparasite of *Colossoma macropomum*	In vitro	All essential oils showed 100% anthelmintic efficacy within 24 h	[124]
100% mortality was observed in the group treated with 100 mg/mL of herbal complex	*Haemonchus contortus*	In vitro	Anthelmintic potential	[125]

## Data Availability

Not applicable.
